# Comparative Evaluation of Biochemical Changes in Tomato (*Lycopersicon esculentum* Mill.) Infected by *Alternaria alternata* and Its Toxic Metabolites (TeA, AOH, and AME)

**DOI:** 10.3389/fpls.2016.01408

**Published:** 2016-09-22

**Authors:** Mukesh Meena, Andleeb Zehra, Manish K. Dubey, Mohd Aamir, Vijai K. Gupta, Ram S. Upadhyay

**Affiliations:** ^1^Department of Botany, Institute of Science, Banaras Hindu UniversityVaranasi, India; ^2^Molecular Glycobiotechnology Group, Discipline of Biochemistry, School of Natural Sciences, National University of Ireland GalwayGalway, Ireland

**Keywords:** *Alternaria alternata*, pathogen, phytotoxic metabolites, toxins, Polymerase chain reaction (PCR)

## Abstract

In the present study, we have evaluated the comparative biochemical defense response generated against *Alternaria alternata* and its purified toxins viz. alternariol (AOH), alternariol monomethyl ether (AME), and tenuazonic acid (TeA). The necrotic lesions developed due to treatment with toxins were almost similar as those produced by the pathogen, indicating the crucial role of these toxins in plant pathogenesis. An oxidative burst reaction characterized by the rapid and transient production of a large amount of reactive oxygen species (ROS) occurs following the pathogen infection/toxin exposure. The maximum concentration of hydrogen peroxide (H_2_O_2_) produced was reported in the pathogen infected samples (22.2-fold) at 24 h post inoculation followed by TeA (18.2-fold), AOH (15.9-fold), and AME (14.1-fold) in treated tissues. 3,3′- Diaminobenzidine staining predicted the possible sites of H_2_O_2_ accumulation while the extent of cell death was measured by Evans blue dye. The extent of lipid peroxidation and malondialdehyde (MDA) content was higher (15.8-fold) at 48 h in the sample of inoculated leaves of the pathogen when compared to control. The cellular damages were observed as increased MDA content and reduced chlorophyll. The activities of antioxidative defense enzymes increased in both the pathogen infected as well as toxin treated samples. Superoxide dismutase (SOD) activity was 5.9-fold higher at 24 h post inoculation in leaves followed by TeA (5.0-fold), AOH (4.1-fold) and AME (2.3-fold) treated leaves than control. Catalase (CAT) activity was found to be increased upto 48 h post inoculation and maximum in the pathogen challenged samples followed by other toxins. The native PAGE results showed the variations in the intensities of isozyme (SOD and CAT) bands in the pathogen infected and toxin treated samples. Ascorbate peroxidase (APx) and glutathione reductase (GR) activities followed the similar trend to scavenge the excess H_2_O_2_. The reduction in CAT activities after 48 h post inoculation demonstrate that the biochemical defense programming shown by the host against the pathogen is not well efficient resulting in the compatible host-pathogen interaction. The elicitor (toxins) induced biochemical changes depends on the potential toxic effects (extent of ROS accumulation, amount of H_2_O_2_ produced). Thus, a fine tuning occurs for the defense related antioxidative enzymes against detoxification of key ROS molecules and effectively regulated in tomato plant against the pathogen infected/toxin treated oxidative stress. The study well demonstrates the acute pathological effects of *A. alternata* in tomato over its phytotoxic metabolites.

## Introduction

Tomato (*Lycopersicon esculentum*) is one of the most important widely grown vegetable crop all over the world (Abd-El Kareem et al., 2006). However, the global economic productivity of this crop is constrained by several phytopathogens. Leaf spot disease of tomato caused by *Alternaria* species is one of the most important disease causing significant losses, and reducing both the food quality and quantity, thus deteriorates the nutritive values of tomato. A wide range of economically important crops including various horticultural, ornamental, and weed species are successfully hosted by *Alternaria* species (Chung, 2012). Tomato plants are infected by the pathogen throughout the entire course of their growth and development, characterized by chlorosis and necrotic symptoms reducing both the food quality and quantity that leads to severe economic losses (Gilchrist and Grogan, 1976).

*Alternaria* species are omnipresent in the environment and produce specific mycotoxins like alternariols [alternariol (AOH), alternariol monomethyl ether (AME), and altenuene (ALT)], tentoxin (TEN), and tenuazonic acid (TeA) (Ostry, 2008). A widespread natural occurrence of these toxins has been reported in various fruits and vegetables as well as their derived products, such as juices, beverages, sauces, and concentrates (Ackermann et al., 2011; Asam et al., 2011, 2013). Exposure to these toxins causes genotoxic, mutagenic, carcinogenic, and cytotoxic effects on both human and animals (Pavón et al., 2012). The toxins AME and AOH have been found in sorghum, sunflower seeds, barley, wheat, oats, tomatoes, mandarin oranges, pepper, and melons which are infected with *A. alternata* (da Motta and Soares, 2000). The toxicogenic potency of *Alternaria* species varies between different isolates, as some isolates have been reported to produce all the three alternariols toxins (AOH, AME, and ALT) in variable quantities (Garg and Singh, 2016). Further, the capacity of a particular isolate to produce different mycotoxins is regulated by their genetic makeup and the amount of mycotoxins produced is well affected by cultural and environmental conditions (Garg and Singh, 2016).

It has been reported that the production of different toxins by necrotrophic fungus *A. alternata* is required for its pathogenesis (Chung, 2012) and after successful infection the host plants induces cell membrane damage (disrupting cell wall proteins), production of ROS molecules and increased H_2_O_2_ accumulation followed by cell death. These ROS molecules brought severe changes in membrane architecture by modifying the chemical groups involved such as lipid peroxidation (Heiser et al., 1998). ROS molecules play an important role in a plethora of mechanism that ranges from plant defense to developmental processes and is of specific importance in host-pathogen relationships as in some cases it was found to be associated directly or indirectly with the development of disease resistance against pathogens (Bandyopadhyay et al., 1999). Moreover, the increased production of ROS molecules affect the physiological and developmental aspects of the infected hosts as characterized by the increased damages to membrane (lipid peroxidation), proteins, carbohydrate, nucleic acids, and pigments such as chloroplasts and/or carotenoids (Das and Roychoudhury, 2014) with decreased seed viability, root growth and increased leaf abscission. Although these ROS molecules are toxic to host plants they also function as signaling molecule and have been demonstrated to be involved in the induction of plant defense genes encoding pathogenesis-related (PR) proteins, genes regulating the accumulation of phenylpropanoid compounds (Schenk et al., 2000). The pathways that regulate ROS homeostasis are crucial for mitigating the toxicity of ROS and provide strong evidence about specificity in ROS signaling (Autréaux and Toledano, 2007). In response to these increased ROS molecules, plants develop a well protective antioxidative network that consists of both non-enzymatic and enzymatic H_2_O_2_ scavengers. Under normal conditions in healthy plants, the antioxidant machinery of the cell is well adapted to minimize the perturbations caused due to ROS molecules. When ROS generation overcomes the cellular antioxidants, the outcome is oxidative stress (Shereefa and Kumaraswamy, 2016). Hypersensitive response (HR) is one of the most effective defense mechanisms developed by plants against their pathogens and characterized by necrosis of the infected tissues to prevent dissemination of the pathogen at nearby cells. Several PR proteins involve in the process and synthesis of antimicrobial compounds such as phytoalexins (Agrios, 2005). Rapid generation of superoxide and accumulation of H_2_O_2_ is the characteristic feature of the HR following the perception of virulent pathogen signals. Emerging data indicates that the oxidative burst reflects activation of a membrane-bound NADPH oxidase (Lamb and Dixon, 1997), which produces (O_2_^∙-^) at the apoplast, and dismutates to H_2_O_2_ spontaneously by the action of SOD (Chung, 2012). The susceptibility of a plant to the pathogen-generated oxidative stress is well determined by the balance between ROS production and its detoxification by the antioxidative defense machinery of the hosts (Sharma et al., 2012). The imbalances found between ROS production and its scavenging by antioxidative enzymes in leaves may reflect defense strategy shown by plants or develop into pathogen success. Defense enzymes such as SOD, CAT, GR, glutathione-*S*-transferase (GST), APx, guaiacol peroxidase (GPx), and peroxidases (POx), as well as some non-enzymatic antioxidants such as glutathione (GSH), tocopherols, carotenoids, ascorbate, flavonoids, proline, and other phenolic compounds (Gill and Tuteja, 2010; Sharma et al., 2012; Kapoor et al., 2015) as it was reported that the activities of defense-related enzymes found to increase several folds during environmental stresses (Jaiswal et al., 2013). CAT, play an important role in detoxifying the activated oxygen species (Sharma et al., 2012) and breakdown peroxide molecules to oxygen and water (Das and Roychoudhury, 2014). APx works more efficiently than CAT in scavenging H_2_O_2_ produced under stressed conditions and is reported to be most widely distributed antioxidant enzyme in plant cells (Sharma et al., 2012). GR catalyses the NADPH dependent reaction of disulphide bond of GSSG and is thus important for maintaining the GSH pool. The phytotoxic metabolites produced by the pathogenic fungus during its interaction with hosts, induce the activation of defense machinery of plants (Huang, 2001), which terminates into the gene expression of various defense-related enzymes. The variant pattern of isoenzymatic profile relevant to antioxidant defense-related proteins clearly demarcate the cellular defense against oxidative stress (Lubaina and Murugan, 2013).

Significant information till to date that evaluates the host response against both the pathogen and its separated toxic metabolites are not available. Therefore, the present study was carried out to examine the comparative physiological and biochemical changes induced by phytopathogenic *A. alternata* and its toxins (TeA, AOH, and AME) during pathogenesis in tomato plants.

## Materials and Methods

### Collection and Culture of Fungal Isolates

Leaf samples from infected tomato plants were collected and then surface sterilized with 0.5% sodium hypochlorite solution for 1-2 min, followed by washing with sterile distilled water (SDW) up to 2-3 times and then grown for 3-4 days on PDA culture medium amended with streptomycin. These samples were incubated at 28°C in dark or light on a 12 h light/dark photoperiod for 6-10 days (Safari Motlagh and Kaviani, 2008). PDA slants made with agar were used for preservation of cultures.

### Identification of the Pathogen

The pathogen was preliminarily identified on the basis of morphological characteristics including size, shape, and structure of conidia and further confirmed by ITS amplification using primers AAF2 and AAR3 amplifying ITS regions and 5.8S genes encoding for *A. alternata.* DNA extraction was carried out as per the method suggested by Doyle and Doyle (1987). The lyophilized mycellar mats of 0.5 g was grinded in a mortar and pestle using 10 ml of CTAB extraction buffer and then incubated at 65°C in a water bath for 30 min. The sample was then mixed with an equal volume of chilled chloroform/isoamyl alcohol and gently mixed followed by centrifugation at 10,000 rpm for 10 min at 4°C temperatures. The supernatant thus obtained was mixed with equal volume of isopropanol and left it for 2 h at 4°C. The sample was again centrifuged at 10000 rpm for 10 min at 4°C temperature. The pellet was then rinsed with 70% ethanol and air dried for 4 h in order to remove the traces of alcohol. Amplification ITS rDNA reaction were performed in 25 μl reaction mixture containing 2.5 μl 10X reaction buffer, 5 μl of each deoxyribonucleotide triphosphate (dNTP), and 1.0 μl each of ITS and 5.8S region specific forward primer AAF2 (5′-TGCAATCAGCGTCAGTAACAAAT-3′) and reverse primer AAR3 (5′-ATGGATGCTAGACCTTTGCTGAT-3′), 0.3 μl of Taq DNA-polymerase and 10-100 ng DNA, 2.5 μl MgCl_2_. The optimized thermal profile of PCR was initial denaturation at 95°C for 3 min, denaturation at 95°C for 30 s, annealing at 70°C for 30 s and final extension at 72°C for 1 min with additional 40 cycles. The amplification was confirmed on a 1% agarose gel in 0.5x TBE buffer and visualized under UV-transilluminator.

### Pathogenicity Test

#### On Plants Leaves

Tomato seeds were scarified in sodium hypochlorite, rinsed in tap water, and then air dried. Plants were grown separately in pots. Physiological conditions such as temperature and humidity for plant growth were maintained at 28° to 32°C and 40 to 60% relative humidity, respectively. Optimum inoculums concentrations were maintained and sprayed were 0.5 × 10^6^, 1 × 10^6^, 1.5 × 10^6^, and 2 × 10^6^ spores/ml, respectively, on leaf area. Plants under experiment were maintained in dew chambers for 8 h at 25°C. These plants were regularly monitored for 2-3 days for disease development and percent control was also determined.

#### On Plants Detached Leaves

The leaves of tomato plants were detached, washed in water, kept in the moist tray and spore suspensions of different isolates of *Alternaria* were prepared. These *Alternaria* isolates were isolated from different plant parts and different locations. To study pathogenicity tomato leaves were taken in a tray along with one as a control that was arranged in a line and placed in a dew chamber for 8 h at 25°C. Inoculum concentration of (2 × 10^6^ spores/ml) was sprayed on each of the detached leaves. The purified toxins were also sprayed separately on the leaves and were regularly monitored for the appearance of pathogenic symptoms, infection severity and disease development.

### Extraction of Metabolite from the Pathogen

Metabolite extraction was carried out using Potato dextrose broth (PDB) as a basal medium. Infectious pathogen was inoculated in the medium and grown for 21 day. Mycelia were separated using a muslin cloth. Filter broth was treated with equal volume of methanol mixed properly and kept at 4°C for 24 h, the precipitate appears were removed and dissolved methanol were evaporated 43°C by the vacuum evaporator. Further remaining broth were extracted with equal volume of ethyl acetate and two immiscible phase (aqueous and organic phase) were separated using a separating funnel. The organic phase contained metabolites which were concentrated at 44°C through vacuum evaporator. Dried crude was dissolved in 1 ml of methanol.

### Purification and Separation of *Alternaria* phytotoxin via Column Chromatography (CC)

Column chromatography (CC) was used to separate *Alternaria* toxins following the methodology as suggested by Devi et al. (2012) with some modifications. A glass column (700 mm **×** 30 mm) was employed for column preparation and silica gel (100-120 mess size Merk) was chosen for the stationary phase. The mobile phase consisted of pure solvent or different solvents as determined by required conditions. For separation of toxic metabolites mobile phase consisted of chloroform: methanol (80:20 and 95:5), benzene: acetone: acetic acid (60:35:5) ratio following gradient elution. The column was loaded with crude metabolites of *A. alternata*. Different fractions eluted from CC were separated by thin layer chromatography (TLC) and further confirmed by the HPLC analysis.

### HPLC-UV Analysis

#### Preparation of the Standard and HPLC Conditions

The three mycotoxins namely TeA, AOH and AME were isolated from pathogen following the protocol as suggested by Andersen et al. (2006) with some minor modifications. Further confirmation was done by HPLC-UV analysis. For HPLC-UV analysis crystallized form of TeA (cat No: T1952), AOH (cat No: A4675), and AME (cat No: A4678) were brought from Biogenuix (LKT laboratories, Inc., New Delhi, India). HPLC calibration was done using a stock solution (1000 μg ml^-1^) and working solution of (10 μg ml^-1^) of toxins kept at -20°C. Chromatographic separations for HPLC was done using base deactivated (250 mm long × 4.6 mm, 5.0 μm particle size) C18 Waters Spherisorb, ODS2 column (product No: PSS831915, USA) having UV-VIS detector (2998 PDA) and Waters 600E system controller. Samples were injected using a 10 μl loop of Waters 717plus autosampler (Waters Corporation, Milford, USA). The mobile phase contained 75% HPLC grade methanol (solvent A) 25% of an aqueous solution (solvent B) of 0.1 M phosphate buffer [Na_2_HPO_4_ (1 M) 7.9 ml + NaH_2_PO_4_ (1M) 92.1 ml dissolved in 900 ml HPLC grade distilled water maintained at pH 5.8 and peak absorbance was monitored at the range of 200-400 nm and a particular wavelength at 254 nm.

#### Estimation of Chlorophyll Content

In tomato plant, disease development was also assessed by observing the chlorophyll content (Chl *a*, Chl *b*, and total chlorophyll). In this method, tomato plants leaves (0.1 g) that were infected with pathogen, and treated with the toxic metabolites of *Alternaria* species. Leaves extracted from both infected/treated samples were chopped into small pieces and extracted with 80% acetone. Chlorophyll contents were estimated by measuring the absorbance at 645 and 663 nm for chlorophyll *a, b*, and total chlorophyll.

Then chlorophyll *a, b*, and total chlorophyll were further calculated according to the Lichtenthaler and Wellburn (1983) formulae.

Chl *a* (mg g^-1^ leaf fresh weight) = [12.7(OD663) – 2.69 (OD645)] × V/1000 × W
Chl *b* (mg g^-1^ leaf fresh weight) = [22.9(OD645) – 4.68 (OD663)] × V/1000 × W
Total Chl (mg g^-1^ leaf fresh weight) = [20.2(OD645) – 8.02(OD663)] × V/1000 × W

(Where, OD = Optical Density, V = Volume of sample, W = Weight of sample).

### Biochemical Profile

#### Cell Death Assay by Evans Blue Staining

Examination and evaluation of the degree and extent of cell death was determined by Evans blue staining, as described by Baker and Mock (1994). For this method the plants were infected with the pathogen and challenged with metabolites. After 48 h, the affected leaves were boiled for 1-2 min in a freshly prepared solution of phenol: lactic acid: glycerol: distilled water (1:1:1:1) contained 20 mg/ml Evans blue stain. After that the tissues were clarified overnight in a solution of 2.5 gm/ml chloral hydrated water. Cell death was observed in the light microscope.

#### DAB Staining to Detect Hydrogen Peroxide (H_2_O_2_) Accumulation

H_2_O_2_ accumulation was detected using histochemical DAB staining as described by Thordal-Christensen et al. (1997). For this staining, the plants were infected with the pathogen and challenged with metabolites. After 48 h, the 1 cm infected leaves were cut with a sterilized blade above the base of petiole and placed in a beaker containing 1 mg/ml DAB–HCl (pH 5.6). Then the leaves were incubated in a humid growth chamber for 12 h (overnight) in a dark place. H_2_O_2_ reacts with DAB to form a reddish-brown stain, this stain was further removed by 96% boiled ethanol to see the accumulation of H_2_O_2_ in the tested leaves, then examined via a light microscope.

#### Quantification of H_2_O_2_

H_2_O_2_ quantification was done to see the effect of the pathogen and metabolites on a tomato plant. For this 0.1 g of leaf sample was taken from each of the treatment was homogenized in an ice bath with 2.0 ml of 0.1% (w/v) of TCA. The homogenate was centrifuged at 12,000 × *g* for 15 min and 0.5 ml of the supernatant was mixed with 10 mM potassium phosphate buffer (pH 7.0) and 1 ml of potassium iodide solution and incubated for 5 min. The oxidation product formed was measured at 390 nm (Velikova, 2000). The amount of H_2_O_2_ formed was determined from the standard curve made with known concentrations of H_2_O_2_ and expressed as nmol H_2_O_2_ g^-1^ fresh weight (FW).

#### Lipid Peroxidation (LPO)

MDA content reveals the amount of total lipid peroxidation and was determined by the thiobarbituric acid (TBA) reaction. The assay was carried out by the method described by Ohkawa et al. (1979). A leaf sample (0.1 g) from each of the treatment was homogenized and incubated with 2.0 ml of 20% TCA (w/v) containing 1% TBA (w/v) for 30 min at 95°C. The reaction was stopped by placing the samples on ice for 10 min and then centrifuged the samples at 10,000 rpm for 15 min. Reaction product absorbance was measured at 532 nm and the amount of MDA was expressed as μmol MDA g^-1^ fresh weight.

#### Antioxidant Enzyme Activities

##### Superoxide dismutase (SOD, EC 1.15.1.1) activity

Superoxide dismutase activity analyzed by determining its ability to prevent the photochemical reaction of nitro blue tetrazolium (NBT) via the method of Beauchamp and Fridovich (1971). Treated tomato plant samples (0.1 g) were crushed in 5 ml ice cold extracting buffer which contained 0.1 M phosphate buffer (pH 7.5) and 0.5 mM EDTA, centrifuged at 15,000 rpm for 15 min and used for enzyme extract. The 3 ml reaction mixture contained 50 mM phosphate buffer, pH 7.8, 13 mM methionine, 75 μM NBT, 60 μM riboflavin, 0.1 mM EDTA, 100 μl enzyme extract and 2 μM riboflavin. Reactions were carried out at 25°C under a fluorescent tube for ten min in an incubator. One unit of SOD activity was defined as the amount of enzyme required to cause 50% inhibition of the rate of NBT reduction measured at 560 nm.

##### Ascorbate peroxidase (APx, EC 1.1.11.1) activity

Ascorbate peroxidase activity was measured according to the method of Nakano and Asada (1981). The reaction mixture consisted of 0.2 ml enzyme extract, 25 mM phosphate buffer (pH 7.0), 0.1 mM EDTA, 0.25 mM ascorbic acid, and 1.0 mM H_2_O_2_. A decrease in absorbance was recorded 60 s after addition of enzyme extract. The oxidation of ascorbic acid was measured at 290 nm and the enzyme activity was expressed as nmol ascorbate oxidized min^-1^ mg^-1^ protein.

##### Catalase (CAT, EC 1.11.1.6) activity

The assessment of CAT activity was determined by using the protocol described by Aebi (1984). For this leaf samples (0.1 g) were homogenized using a chilled mortar and pestle with a solution of 50 mM Tris-HCL buffer (pH 8.0) whose composition comprises of 0.5 mM EDTA, 2% w/v polyvinylpyrrolidone, and 0.5% (v/v) Triton X100. The homogenate was centrifuged at 15,000 × *g* for 10 min at 4°C, and the supernatant obtained was used for further assessment of enzymatic activity. For this 1 ml enzyme extract was dissolved in 300 μM phosphate buffer (pH 7.2) amended with 100 μM H_2_O_2_. Enzymatic activity was determined by measuring the total oxygen released from enzymatic dissociation of H_2_O_2_ in darkness for 1 min. The evolution of oxygen was estimated by measuring the decrease in H_2_O_2_ absorption at 240 nm and enzyme activity was enumerated as nmol min^-1^ mg^-1^ protein.

##### *Glutathione reductase* (GR, EC 1.6.4.2) activity

For the assessment of GR activity a method adopted by Anderson (1996) was employed. In this leaf samples (0.1 g) were homogenized using a chilled mortar and pestle in 5 ml of 50 mM Tris-HCl buffer (pH 7.6). The homogenate was centrifuged at 15,000 × *g* for 30 min at 4°C, and the supernatant obtained was used further for the determination of enzymatic activity. The reaction mixture comprises 50 mM Tris-HCl buffer (pH 7.6), 10 ml NADPH (0.15 mM), 100 μl oxidized glutathione (1 mM GSSG), 3 mM MgCl_2_, and 0.3 ml enzyme extract. GR activity was assayed by measuring the decrease in absorbance of NADPH at 340 nm and the activity of the enzyme was expressed as NADPH oxidized μmol min^-1^ mg^-1^ protein.

#### Protein Preparation for SDS-polyacrylamide gel electrophoresis (PAGE)

Leaf samples (200 mg) of treated tomato plants were harvested and protein was extracted by freeze dried sample grinding with mortar and pestle in liquid nitrogen followed by extraction with 2 ml extraction buffer solution (250 mM sucrose, 25 mM Tris, pH 7.2). The samples were centrifuged for 10,000 × *g* for 20 min. After which protein was quantified by using the method of Lowry et al. (1951) taking bovine serum albumin (BSA, Sigma) as standard. Further 40 μg of protein were subjected to SDS-polyacrylamide gel electrophoresis (PAGE) according to the method of Laemmli (1970).

#### Isoenzyme Profile of SOD

A modified method of Jaiswal et al. (2013) was used for isoenzyme profiling of SOD. Native PAGE was carried out by 10% polyacrylamide gels. To visualize enzyme isoforms same concentration (40 μg) of protein were loaded for control and treated sample, after an electrophoretic run at 4°C, the gels were incubated at room temperature for 15 min in the dark in a mixture containing 12.5 mg NBT and 5 mg riboflavin dissolved in 50 ml Tris-HCL buffer (pH 8.2). The riboflavin and NBT solution were removed and added 10 ml (0.1%) TEMED again incubated in dark for 15 min. Removed the solution and light induced the enzyme isoforms.

#### Isoenzyme Profile of CAT

Changes in isoform and expression of CAT in tomato plants were performed according to the method of Davis (1964) by using native PAGE. Tris-glycine (pH 8.3) was used as electrode buffer, 3.5% stacking and 7.5% running gels were used. Enzyme samples containing 30 μg protein, and mixed with glycerol were applied on top of the stacking gel and electrophoresis was performed using a current of 25 mA. Gels were soaked in 5 mM K-phosphate buffer (pH 7.0) and then transferred to a 5 mM H_2_O_2_ solution in the same buffer, for detection of CAT isoforms. Gels were rinsed with water and stained in a reaction mixture consisting of 2% (w/v) potassium ferricyanide and 2% (w/v) ferric chloride, after 10 min incubation. The isoenzyme bands appeared colorless in the deep blue background.

### Statistical Analysis

The data presented are means ± standard deviation (SD) for independent experiments of all treatment. One-way ANOVA followed via Duncan’s multiple range test at the *P* ≤ 0.05 significance level, carried out using SPSS (IBM SPSS Statistics version 20) software package for expressing the statistical significance.

## Results

### Isolation and Identification of the Pathogen

**FIGURE 1:**
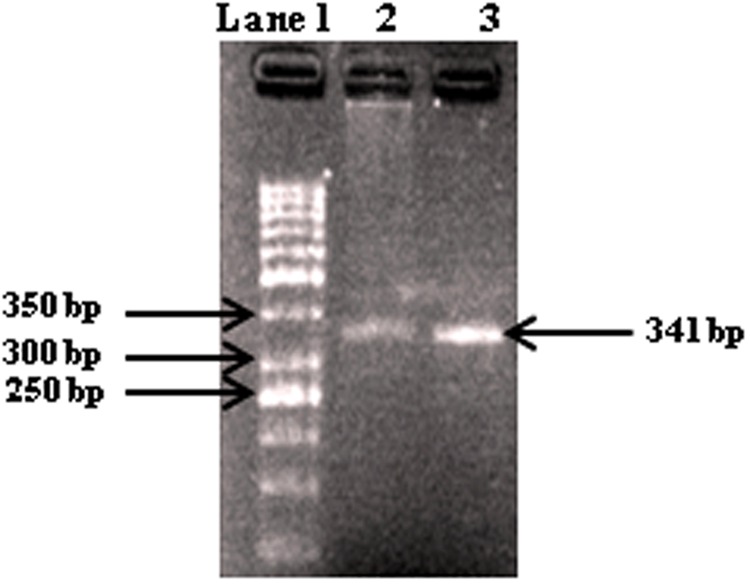
**Gel electrophoresis of PCR products with primers AAF2/AAR3 of DNA from fungal isolates.** Lane 1, molecular weight markers (1 kb ladder); lanes 2-3, *Alternaria alternata* (341 bp).

On the basis of preliminary microscopic examination based on morphological characteristics the pathogen was identified as *A. alternata.* This was further confirmed on the basis of pathogen-specific primers (approximately 25 bases in length) coding ITS region and 5.8S gene having some regions specific for the pathogen. The size of the amplicon was found at 341 bp characteristic for *A. alternata* (**Figure 1**). Further ITS sequencing was made using universal primers ITS1 (forward) and ITS4 (reverse) and the r DNA sequences thus obtained were analyzed using NCBI-BLAST. The BLAST results confirmed the species as *A. alternata.* The sequence was submitted to gene bank and an accession number KX118413 was assigned for the identified isolate.

### Pathogenicity Test

**FIGURE 2:**
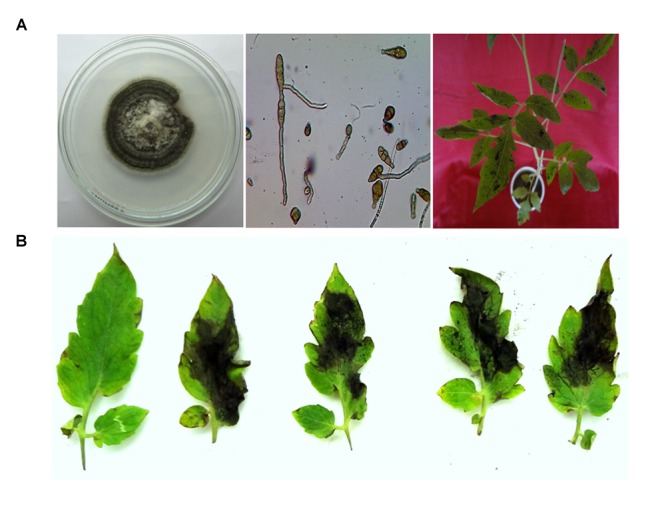
**(A)** Isolation, purification, characterization, and infection of pathogen *A. alternata* on tomato plant. **(B)** Infection on detached leaves of tomato plant by the *A. alternata* and its toxins, showing the area of necrotic lesions and control leaves without necrotic lesions.

Pathogenicity test was performed via Koch’s postulates and resulted in necrotic lesion on leaves produced. Necrotic lesions consist of brown to black sunken with typical concentric rings, which increased regularly and covered almost 3/4 of leaf area within 10-12 days. The isolated pathogen from infected portions showed a similar kind of colony growth and conidial morphology and exhibited pathogenicity for tomato, establishing the Koch’s postulates (**Figure 2A**).

### Infection on Tomato Leaves by Spore Suspension of *Alternaria alternata* and its Toxins

Spore suspensions of the pathogenic isolates were sprayed on the tomato leaves, observation indicated that the disease symptoms were appeared at different time intervals. **Figure 2B** showed infection of a spore suspension of *Alternaria* pathogen on 0, 2, 4, 6, and 8 days. The symptoms of the pathogen were appeared (showing necrosis) on the each leaf except control. Symptoms were appeared as a concentric ring on the leaves after necrosis. In the result pathogenic isolate sprayed on the leaf was highly pathogenic, whereas toxins TeA, AOH, AME challenged leaves were less pathogenic, respectively. Along with the infection, leaves had also lost their chlorophyll contents and became yellow. Infiltration with sterile medium caused no necrosis as comparable to those of deionized water (**Figure 2B**).

### Identification of TeA, AOH, and AME

**FIGURE 3:**
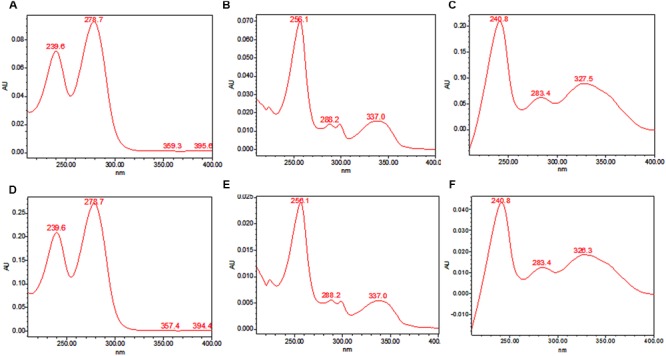
**UV absorption spectra recorded for standard metabolites and those isolated from *A. alternata* using HPLC. (A)** absorption peaks for standard TeA at 239.6 nm and 278.7 nm, **(B)** absorption peaks for standard AOH at 256.1, 288.2, and 337.0 nm, **(C)** absorption peaks for standard AME at 240.8, 283.4, and 327.5 nm, **(D-F)** absorbance peaks as evaluated from metabolites TeA, AOH, and AME extracted from fungal pathogen.

TeA, AOH, and AME were purified by column chromatography and identified by HPLC analysis on the basis of their retention time compared with their standard. Further confirmation was made on the basis of HPLC-UV analysis. The standard peak value for each toxin was determined using HPLC-UV and then the peak values for separated mycotoxins isolated from different isolates of *Alternaria* were compared those with the standard value. The UV absorbance spectra of isolated TeA, AOH, and AME were shown in **Figure 3.** The absorbance peak at 239.6 and 278.7 nm (TeA); 256.1, 288.2, and 337.0 nm (AOH); and 240.8, 283.4, and 327.5 nm (AME) were clearly observed (**Figure 3**). These observed values were found to be same as those were reported for standard values and thus confirms the presence of those toxins.

### Effect on Chlorophyll Content

**FIGURE 4:**
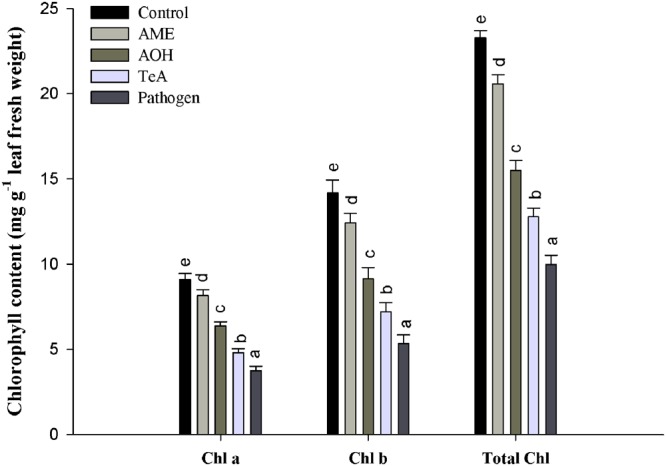
**Effect on chlorophyll content in tomato plants treated with pathogen and their metabolites after 48 h. The results are expressed as the mean of three replicates and vertical bars indicate the ±SD of the mean**.

Chlorophyll (chl *a*, chl *b* and total chlorophyll) content of plant decreased gradually after challenging with toxins (TeA, AOH, and AME) that were isolated from *A. alternata*. It has been observed that minimum chlorophyll content in pathogen treated plants (**Figure 4**) when compared to the control. According to the formulae, as suggested by Lichtenthaler and Wellburn (1983), the maximum reduction (41.2%) in chlorophyll *a* content was observed in pathogen-infected samples followed by TeA (52.76%), AOH (69.92%), and AME (87.61%) treated samples compared to untreated control. Similarly, a maximum decrease (37.65%) in chlorophyll *b* content was reported from pathogen infected samples followed by TeA (50.77%), AOH (64.47%), and AME (87.61%) challenged. The total chlorophyll content followed the same pattern and found to be maximally reduced (42.88%) in pathogen treated samples followed by TeA challenged (54.94%), AOH challenged (66.60%) and AME challenged (88.41%). Necrotic spots emerged in leaf tissues were more prominent in pathogen-inoculated samples with a maximum decrease in chlorophyll content followed by toxins AME, AOH, and TeA challenged plants.

### Detection of H_2_O_2_ in leaves

**FIGURE 5:**
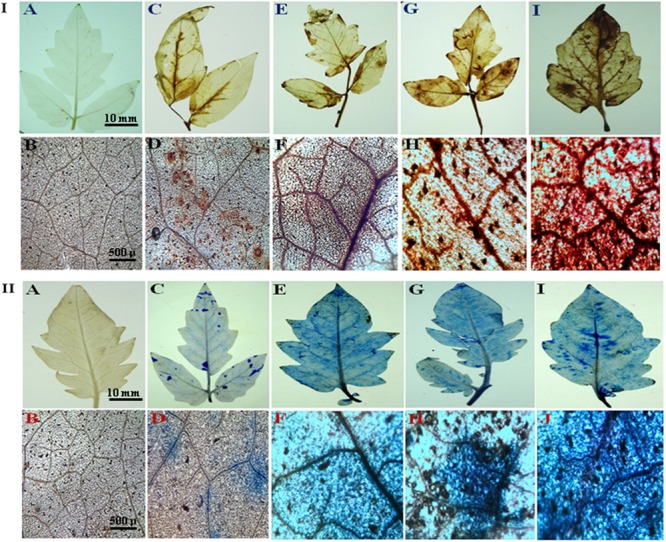
**(I)** Hydrogen peroxide production in tomato leaves as visualized by DAB staining. (A) control leaf; (B) microscopic view; (C) AME treated leaf; (D) microscopic view; (E) AOH treated leaf; (F) microscopic view; (G) TeA treated leaf; (H) microscopic view; (I) pathogen infected leaf; (J) microscopic view. **(II)** Cell death assay by Evans blue staining. (A) control leaf; (B) microscopic view; (C) AME treated leaf; (D) microscopic view; (E) AOH treated leaf; (F) microscopic view; (G) TeA treated leaf; (H) microscopic view; (I) pathogen infected leaf; (J) microscopic view.

H_2_O_2_ production was visualized as a reddish brown stain by DAB, and was more prominent in pathogen infected plant samples (**Figure 5I**). Quantitative measurements of H_2_O_2_ content were also studied in tomato plants before and after pathogen infection along with their toxins treatment. Significant changes were observed in the toxins treated plants both before and after challenge, but the response was stronger and more visible in the pathogen infected plants.

### Cell Death Assay by Evans Blue Staining

The cell death assay was evaluated by staining with Evans blue dye. It is a non-toxic, water-soluble dye commonly used to selectively stain dead cells. Pathogen infected tissues showed intense blue coloration of the dye indicating more cell death in pathogen treated samples. The intensity of blue coloration was more prominent in TeA treated tissues but less than pathogen treated samples, followed by decreased intensities in AOH and AME treated samples (**Figure 5II**). The maximum cell death was reported in pathogen infected samples whereas control plant samples remained unstained.

### H_2_O_2_ Estimation

**FIGURE 6:**
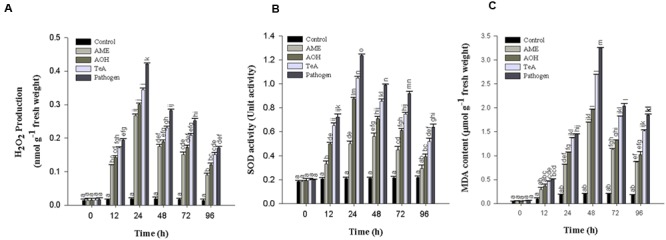
**(A)** Production of Hydrogen peroxide (H_2_O_2_)_,_ **(B)** Superoxide dismutase (SOD), and **(C)** Lipid peroxidation (MDA content) in tomato plant at different time intervals infected by *A. alternata* and treated with its toxins. The results are expressed as the mean of three replicates and vertical bars indicate the ±SD of the mean.

During pathogen inoculation the amount of H_2_O_2_ produced increases. In our result, a sharp increase in the amount of H_2_O_2_ produced was reported at 12 h post inoculation which increased gradually and reached maximum at 24 h (**Figure 6A**) followed by a continuous decrease in all treated samples. The H_2_O_2_ concentration was reported to be maximum (22.2-fold) in pathogen infected samples followed by TeA (18.2-fold), AOH (15.9-fold) and AME (14.1-fold) treated samples as compared to control plant samples.

### Lipid Peroxidation Assay

The lipid peroxidation profile was assessed with MDA content as the level of MDA was found to be increased gradually in the leaves of pathogen infected samples. The LPO activity was reported to be highest at 48 h post inoculation in the extracted leaves and thereafter decreases upto 96 h (**Figure 6C**). The LPO activity at 48 h post inoculation was reported to be maximum in pathogen-infected samples (15.8-fold) followed by TeA (13.0-fold), AOH (9.5-fold), and AME (8.2-fold) treated samples as compared to control plant samples.

### Antioxidant Enzyme Activities

#### Superoxide Dismutase Activity

Superoxide dismutase activity first increases from 0 to 24 h after pathogen inoculation and /or exposure of toxins to the leaf tissues and then decreases successively. It was reported to be higher in all treated samples at 24 h post inoculation. The maximum activity was reported in pathogen infected samples (5.9-fold), followed by TeA (5.0-fold), AOH (4.1-fold), and AME (2.3-fold) treated samples compared to non-inoculated and unchallenged control samples (**Figure 6B**).

#### Ascorbate Peroxidase Activity

**FIGURE 7:**
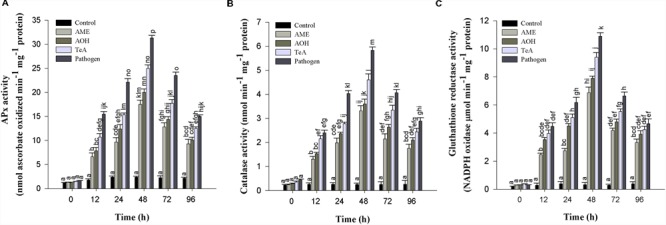
**(A)** Ascorbate peroxidase (APx), **(B)** Catalase (CAT), and **(C)** Glutathione reductase (GR) enzymatic activity in tomato plant at different time intervals infected by *A. alternata* and treated with its toxins. The results are expressed as the mean of three replicates and vertical bars indicate the ±SD of the mean.

Ascorbate peroxidase has much higher affinity for H_2_O_2_ than CAT which revealed that APx is efficient scavengers of H_2_O_2_ under stressful conditions. The APx activity first increases from 0 to 48 h after pathogen inoculation and/or toxins treatment thereafter decreased successively up to 96 h. Pathogen infected samples showed highest APx activity (13.1-fold) followed by TeA (10.4-fold), AOH (8.3-fold), and AME (7.3-fold) treated samples as compared to control plant samples (**Figure 7A**).

#### Catalase Enzyme Assay

Catalase activity in the tomato plants were also increased from 0 to 48 h and then decreased up to 96 h in toxins and pathogen treated plants. The maximum activities were seen in pathogen infected plants (23.0-fold) higher, followed by toxin TeA (18.1-fold) which then follows AOH (14.2-fold) and AME (13.0-fold) treated samples as compared to control samples (**Figure 7B**).

#### Glutathione Reductase

Glutathione reductase activity in tomato plants was found to increase at 48 h and then deceased up to 96 h. The activity was found to be maximum at 48 h for pathogen infected plant samples (33.4-fold), followed by toxin TeA (28.8-fold) and AOH (24.2-fold) and AME (21.0-fold) treated samples as compared to control (**Figure 7C**).

#### Protein Estimation

**FIGURE 8:**
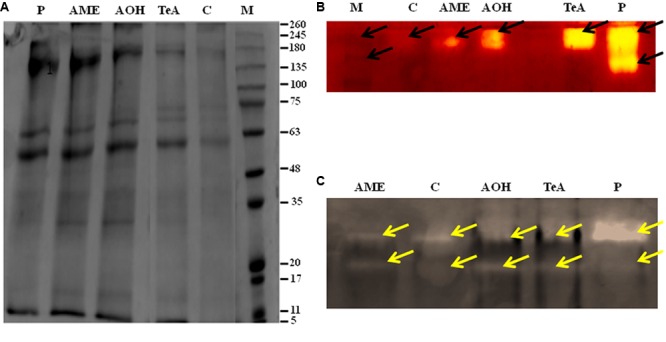
**(A)** Protein pattern (SDS-PAGE) of soluble protein extracted from stressed tomato plants leaves infected by *A. alternata* and treated with its toxins as compared to the control. Arrows indicate the increase and decrease in intensity after infection. Isoenzyme profile of **(B)** Superoxide dismutase (SOD), and **(C)** Catalase (CAT) infected by *A. alternata* and treated with its toxins.

The protein profile was studied by using SDS-PAGE technique. Total proteins from both pathogen infected and metabolite challenged plants were extracted. The approximate molecular weight of the isolated proteins were determined by using marker proteins of medium range. The approximate molecular weight of the polypeptide ranged from 11 to 245 kDa. The result showed significant variation in the protein profile as total 12 bands were observed in pathogen infected samples, 14 bands in AOH treated samples and 15 bands were present in AME treated samples at 48 h post inoculation as determined by using gel documentation unit (**Figure 8A**). Protein profile studies showed the occurrence of common bands of 118.1, 68.5, 54.2, 45.2, 34.2, 27.9, 22.6, and 12.3 kDa in all treated samples (**Figure 8A**). In contrast, the pathogen treated samples showed a unique band of ∼147.8 kDa. Whereas two protein bands of ∼101.7 and ∼86.6 kDa were found to be reported in each of TeA, AOH, and AME treated samples. This differential protein profile indicates the expression of different proteins by the host during pathogen inoculation and /or toxic induced challenges.

#### Isoenzyme of SOD

Native PAGE results showed alteration in band intensities for antioxidative defense enzymes. The isozymic profile for SOD activity showed two different bands of size 58 kDa (SOD1) and 47 kDa (SOD2) (**Figure 8B**). This alteration in band intensities was found to be more significant in pathogen infected samples, showing more intense bands followed by toxic induced samples. This provides evidence regarding the enhanced expression of these enzymes during pathogen inoculation.

#### Isoenzyme of CAT

The isozymic profile of CAT showed two different bands of 72 kDa (CAT1) and 63 kDa (CAT2) of higher intensities were reported from pathogen treated plant samples when compared to toxin treated tissues where bands of light intensities were found. This indicates the substantial rise in CAT activities under pathogenic stress conditions (**Figure 8C**).

## Discussion

The fungal genus *Alternaria* comprises a group of ubiquitous and saprophytic fungi producing toxins (Rotem, 1994). *Alternaria* species have been reported to cause diseases in nearly 400 plant species of which *A. alternata* alone can infect more than 100 plant species (Kusaba and Tsuge, 1995). The successful pathogenicity of this pathogen is due to the presence of diverse toxins having a unique mode of action (Montemurro and Visconti, 1992). Scott (2001) reported that *A. alternata* is a frequently occurring species in field and storage and produces a number of mycotoxins, including alternariols (AOH, AME, and ALT) and TeA. The exposure of pathogen or its toxic metabolites disturb the normal physiological processes like photosynthesis, respiration, translocation, transpiration, growth and development and characterized by major biochemical changes that include the synthesis of antioxidative defense enzymes and accumulation of specific metabolites (Taj et al., 2015). Pathogen or toxins induced cell damages could be manifested in the form of specific symptoms including wilting, growth suppression, chlorosis, necrosis, and spotting of aerial portions. In our results, the maximum decrease in chlorophyll *a*, chlorophyll *b*, and the total chlorophyll content was reported in pathogen challenged plant samples followed by TeA, AOH, and AME treated samples. The necrotic spots produced on pathogen infected samples were more prominent than toxin treated tissues indicating the pathogen and/or toxin induced malfunctioning of photosynthetic machinery followed by cell death.

The oxidative burst or rapid and transient production of a large amount of ROS belongs to the fastest and earliest active defense responses to a microbial infection known in plants (Lubaina and Murugan, 2013). In the present investigation, we reported that the amount of H_2_O_2_ and superoxide anion (O_2_^-^) accumulated was found to be increased significantly after the successful recognition of pathogen or its toxic metabolite. Therefore, high accumulation of H_2_O_2_ and O_2_^-^ represent the process of disease resistance mechanism involved either directly or indirectly to prevent the pathogen growth and dissemination and to activate more aggressive form of defense reactions in that interval. This was also confirmed on the basis of histochemical studies using DAB staining as the red-brown coloration showed the possible sites of H_2_O_2_ accumulation and extensive ROS activity leads into cell death which was confirmed by Evan blue dye staining and it was reported that this dye can be easily absorbed by dead cells, which demonstrate that pathogen and their metabolites induced cell death in treated tissues. El-Khallal (2007) reported that increased accumulation of H_2_O_2_ may lead to accelerated senescence and decreased photosynthetic rate. H_2_O_2_ is independently involved in the modification of plant cell walls through peroxidase-catalyzed cross-linking of polymers, such as proteins (Brisson et al., 1994). Shimizu et al. (2006) have demonstrated the effect of AK-toxin I on host cells and reported that the generation of H_2_O_2_ was maximum in membrane fragment and the plasma membrane of toxin-treated sensitive tissues. The lipid peroxidation was probably induced by ROS in the modified plasma membrane of toxin-treated sensitive tissues. ROS was proposed to act synergistically in a signal amplification loop with shikimic acid-dependent pathways to drive the HR and the establishment of systemic defense (Lubaina and Murugan, 2013). Our results showed that the extent of lipid peroxidation at 48 h post inoculation was maximum (15.8-fold) in pathogen-inoculated leaf samples followed by TeA (13.0-fold), AOH (9.5-fold) and AME (8.2-fold) treated sample compared to control, suggesting that the biochemical defense pathway is more in the favor of pathogen. The elevated concentration of H_2_O_2_ in cellular system positively correlates the oxidative changes affecting MDA content as MDA is the marker for lipid peroxidation released from cellular membranes of tissues and are formed by the reaction of ROS (H_2_O_2_ or/and O_2_^-^) with lipid molecules (Shimizu et al., 2006). Lubaina and Murugan (2013) have reported that infection of *A. sesami* increases the amount of lipid peroxidation in the pathogen inoculated leaf samples with increased MDA content. Several studies revealed that different antioxidative defense enzymes involve protecting the plants during abiotic and biotic stress (Kholova et al., 2009). We have shown that high SOD activity was found in all samples due to the high ROS accumulation as SOD provides the first line of defense against oxidative stress (Bowler et al., 1992) and involved in dismutation of O_2_^-^ to H_2_O_2_ and O_2_ (Treutter, 2006). In our results, SOD activity was found to be maximum in the pathogen treated samples compared to individually treated toxin samples. Ehsani-Moghaddam et al. (2006) reported the high SOD activity in leaves infected by *Mycosphaerella fragariae* and was higher in resistant strawberry cultivars. The increased CAT activity for 0 to 48 h demonstrated the removal of excess H_2_O_2_ produced (Mittler, 2002). The reduction in CAT activity after 48 h is due to the excessive production and accumulation of H_2_O_2_ and this reduction may be due to the enhanced proteolysis caused by peroxisomal endopeptidases which were induced by oxidative stress (Palma et al., 2002). Durner and Klessig (1996) suggested that SA-mediated inhibition of CAT and APx probably results from peroxidative reactions. However, the activities of SOD enzymes was not in coordination with H_2_O_2_ scavenging enzymes (CAT and APx) produced and suggest that decreased CAT activity might be a part of defense reaction to provide resistance against the pathogens or toxins, due to the fact that at decreased CAT activity, plant can tolerate high concentrations of H_2_O_2_. Debona et al. (2012) compared the pathogen induced biochemical changes in resistant (BRS229) and susceptible (BRS18) wheat varieties and found that CAT activity was more prominent in resistant variety which supports the role of CAT in providing disease resistance. The native PAGE result showed the alterations in intensities of isozyme bands in the pathogen treated samples as the CAT and SOD bands were intense followed by reduced intensities for TeA, AOH and AME exposed samples which support our results for increased expression of defense enzymes during stress conditions. Jaiswal et al. (2013) reported the alteration in the isozymic profile of antioxidative defense enzymes in pumpkin following the infection caused by the begomovirus. SDS-PAGE result explains the protein profile of the pathogen treated samples and shows the differential expression of proteins in both the pathogen inoculated samples and the toxins treated samples. This further confirms that these toxins have differential effect on host defense machinery. Further, the increased activities of APx, GR, and other antioxidative enzyme indicate that they play a key role in maintaining the constant level of O_2_^-^ and H_2_O_2_ during the pathogen infection process to provide resistance against pathogens or its toxins thereby limiting the cellular damages caused by these ROS molecules. One more antioxidative pathway that removes the excessive H_2_O_2_ is an ascorbate-glutathione cycle (Asada, 1994) and comprises of series of redox reactions involving enzymes APx, dehydroascorbate reductase (DHAR) monodehydroascorbate reductase (MDHAR) and GR (Nakano and Asada, 1981). APx is responsible for removing excessive H_2_O_2_ from chloroplast, peroxisomes, and mitochondria (Quan et al., 2008) using ascorbate as a specific electron donor to reduce H_2_O_2_ to water (Asada, 1992). It has been well documented that the level of *apx* transcript and enzymatic activity increases during the plant-pathogen interaction (Agrawal et al., 2002). The GR-mediated reaction is NADPH-dependent and catalyzes the reduction of oxidized glutathione to produce its reduced form (Ahmad et al., 2010). In this way, the regenerated reduced glutathione (GSH) maintains the total ascorbate (ASH) pool and thus protects the plant tissues against oxidative stress (Ding et al., 2009). In our result, GR activity was found to be increased 33.4-fold with the pathogen treated samples followed by challenged toxic metabolites. However, the role of GR in plant resistance against pathogens is not well demonstrated, suggesting its role in host resistance. Garcia-Limones et al. (2002) reported the increased activity following the infection caused by a virulent isolate of *Fusarium oxysporum* f. sp *ciceris*. It has been reported that several enzymes have been implicated in apoplastic ROS production following successful pathogen recognition. These results suggest that increased levels of ROS may be contributed by a decrease in antioxidant defense enzymes (decreased scavenging potential) and high ROS accumulation imparts resistant reaction developed by the host. The pathogen success or host wining strategy is decided by an imbalance between H_2_O_2_ generation and scavenging enzymes which may reflect defense mechanism in tomato or a pathogenic strategy of the fungus.

## Conclusion

The study confirms the role of *Alternaria alternata* mycotoxins has phytotoxic effects. The toxin induced biochemical changes evaluates its potency and contribution in disease development. The physiological and biochemical defense response as evidenced by increased activities of defense enzymes and generation of ROS molecules is an important indication of the role of these key molecules in plant-pathogen interactions. Some mycotoxins are host selective and play an important role in fungal virulence and therefore, in disease development. Further studies on these phytotoxins could resolve the better understanding of host-pathogen interactions and could be used as an antibiotic against noxious pathogens. Currently, these phytotoxins are used as probes for rapid screening of plant clones or the progenies from crosses for disease resistance.

## Author Contributions

MM, sample collected, executed most of the experiments and designed the manuscript. AZ, MD, and MA, executed some of the experiments. RU, designed the experiments and strategy of the sample collections. VG, resolution of the critical questions related to the accuracy of the data. All authors read and approved the final manuscript.

## Conflict of Interest Statement

The authors declare that the research was conducted in the absence of any commercial or financial relationships that could be construed as a potential conflict of interest.

The reviewer MR and handling Editor declared their shared affiliation, and the handling Editor states that the process nevertheless met the standards of a fair and objective review.

## Abbreviations

AP_X_: Ascorbate peroxidase
CAT: Catalase
DAB: 3,3′- Diaminobenzidine
H_2_O_2_: Hydrogen peroxide
GR: Glutathione reductase
MDA: Malondialdehyde
PDA: Potato dextrose agar
ROS: Reactive oxygen species
SDS: Sodium dodecyl sulfate
SDW: Sterile distilled water
SOD: Superoxide dismutase
TCA: Trichloroacetic acid
